# Spoonfeeding is associated with increased infant weight but only amongst formula‐fed infants

**DOI:** 10.1111/mcn.12941

**Published:** 2020-01-13

**Authors:** Sara Wyn Jones, Michelle Lee, Amy Brown

**Affiliations:** ^1^ Department of Public Health, Policy and Social Sciences Swansea University Swansea UK; ^2^ Department of Psychology Swansea University Swansea UK; ^3^ Centre for Lactation, Infant Feeding and Translation (LIFT) Swansea University Swansea UK

**Keywords:** breastfeeding, complementary feeding, formula milk, infant length, infant weight, solid foods, baby‐led weaning

## Abstract

Infant feeding experiences are important for the development of healthy weight gain trajectories. Evidence surrounding milk feeding and timing of introduction to solids is extensive; however, the impact of the method of introducing solids on infant growth has been relatively underexplored. Baby‐led weaning (where infants self‐feed family foods) is proposed to improve appetite regulation, leading to healthier weight gain and a reduced risk of obesity. However, the evidence is mixed and has methodological inconsistencies. Furthermore, despite milk being a large part of the infant diet during the period infants are introduced to solid foods, its influence and interaction with introductory style have not been considered. The aim of this study was to explore growth among infants aged 3–12 months according to both style of introduction to solid foods and milk feeding; 269 infants were weighed and measured, and body mass index (BMI) computed. The results showed that overall, infants who were spoon‐fed (compared with self‐fed) at introduction to complementary feeding (CF) had greater length (but not weight or BMI). However, when milk feeding was accounted for, we found that infants who were both spoon‐fed and fully formula fed had greater weight compared with spoon‐fed, breastfed infants. There was no significant difference in weight among self‐fed infants who were breastfed or formula fed. The results highlight the importance of considering infant feeding as a multicomponent experience in relation to growth, combining both milk feeding and method of CF. This relationship may be explained by differences in maternal feeding style or diet consumed.

**Key Messages**

- Early feeding experiences are important in relation to growth and appetite regulation in infancy and later life.
- There is an association between spoon‐feeding and length, but this association does not increase over time, suggesting a longer length may lead to an infant being spoon‐fed.
- Infants who are spoon‐fed and fully formula fed are heavier than those spoon‐fed and breastfed, but no difference in weight is seen for self‐fed babies whether they are breastfed or formula fed.
- A responsive feeding approach is likely to foster healthy weight gain trajectories and appetite regulation. Infants who are both spoon‐fed and bottle fed may have less opportunity to self‐regulate their intake of food.

## 1 INTRODUCTION

Feeding experiences during the first year of life are recognised as playing an important role in laying down the foundations of weight and eating behaviour (Snethen, Hewitt, & Goretzke, 2007). Although research has typically focused on milk feeding (Bartok & Ventura, 2009), increasing recognition is being given to the latter part of infancy, in particular when and how infants are introduced to complementary foods (Brown, Jones, & Rowan, 2017; Daniels, Mallan, Fildes, & Wilson, 2015).

In terms of how infants are introduced to complementary foods, a baby‐led weaning (BLW) approach, where infants self‐feed family foods, has been proposed to promote healthier weight gain trajectories than a traditional spoon‐feeding approach. Research with mothers who follow BLW highlights a perception that infants have greater control over their intake of food and therefore demonstrate greater satiety responsiveness (regulation of food intake), supporting a healthy weight (Arden & Abbott, 2015; Brown & Lee, 2013; Rapley, 2015).

Research examining the impact of a baby‐led approach upon satiety responsiveness is mixed. In one study, infants aged 18–24 months who followed a baby‐led approach to starting solids were rated as more satiety responsive compared with those who had been spoon‐fed (Brown & Lee, 2015). Conversely, in a more recent study, no significant difference was found between weaning approach groups (Komninou, Halford, Harold, 2019), and in a trial of a baby‐led versus standard weaning approach, BLW was actually associated with reduced satiety responsiveness at 24 months (Taylor et al., 2017).

Additionally, research examining the impact of weaning style upon infant and child weight is mixed. In two cross‐sectional studies based in the UK, a BLW approach was associated with a lower weight and reduced incidence of overweight among infants aged 18–24 months (Brown & Lee, 2015) and in preschool children (Townsend & Pitchford, 2012). However, in both studies, mothers who participated were self‐selecting, and both relied on maternal self‐report of infant weight of many of the infants.

Conversely, the New Zealand Baby‐Led Introduction to SolidS (BLISS) study—a trial that randomised mothers to either a baby‐led approach or traditional feeding approach using research‐led clinical measurements of infant growth—found no difference in infant weight at 12 or 24 months. However, adaptations made to the specific baby‐led feeding approach encouraged may differ from how many families practice BLW in the community. Families were encouraged to offer their infants protein at every meal, with an emphasis on good fats, as there was concern that infants may not self‐feed sufficient calories (Taylor et al., 2017).

Infant growth is affected by many factors, and it is unlikely that any one behaviour in insolation will affect weight trajectories. As noted, considerable research has shown the protective impact of breastfeeding upon infant weight. Although differences in milk content partly explain this, through breast milk having a lower protein content alongside bioactive properties that can support appetite regulation (Chan et al., 2017), differences in feeding style have also been identified. Compared with bottle feeding, breastfeeding is associated with a more responsive feeding style (Brown, Raynor, & Lee, 2011), lower intake of milk (Hester, Hustead, Mackey, Singhal, & Marriage, 2012), and greater satiety responsiveness (Brown & Lee, 2012).

Research has not considered how milk feeding and method of introduction of complementary foods might interact to affect weight gain trajectories. In the BLISS trial, breastfeeding rates were higher than average, with New Zealand breastfeeding rates considerably higher than U.K. breastfeeding rates. In the cross‐sectional studies, breastfeeding duration was either used as a control variable (Brown & Lee, 2015; Komninou, Halford, & Harrold, 2019) or not considered (Townsend & Pitchford, 2012).

Milk feeding method has been shown to interact with timing of complementary feeding to affect weight. In the European IDEFICS (Identification and prevention of Dietary‐ and lifestyle‐induced health EFfects In Children and infantS) study, infants who were breastfed throughout the first year had a reduced risk of overweight compared with those who discontinued breastfeeding (Papoustou, Savva, Hunsberger, Jilani,, & Ahrens, 2018). Similarly, in a U.S. birth cohort study of 847 children, infants who were introduced to complementary foods before 4 months had an increased risk of overweight but only if they were formula fed (Huh, Rifas‐Shiman, Taveras, Oken, & Gillman, 2011). This impact has been attributed to bottle‐fed infants being less likely to reduce their intake of milk when complementary foods are added to the diet (Heinig, Nommsen, Peerson, Lonnerdal, & Dewey, 1993; Noble & Emmett, 2006). Therefore, it is likely that milk feeding may interact with method of introducing complementary foods to affect infant growth.

The aim of this study was to conduct the first U.K.‐based study examining the impact of method of complementary feeding and how it interacts with milk feeding upon infant growth during the first year using solely researcher‐led measurements.

## 2 METHODS

### 2.1 Participants

Participants were from the SHIFT (Studying Healthy Infant Feeding Trajectories) study in the United Kingdom, which studied the impact of different aspects of infant feeding upon weight and growth of infants aged 3–12 months in the United Kingdom. Data were collected between February 2016 and November 2017. Participants are mother–infant dyads from South Wales and South West of England. All infants in the study were born >37‐week gestation and > 2,500 g. Infants with significant health concerns or history of weight gain or feeding issues, such as failure to thrive, severe colic, or severe reflux (as reported by the mother), were excluded from participation.

### 2.2 Measures

Infant weight and length were measured by the lead researcher using SECA® calibrated scales and SECA length measuring mat. Mothers self‐reported infant birth weight. Weight‐for‐age *z*‐score (WAZ), length‐for‐age *z*‐score (LAZ), body mass index‐for‐age *z*‐score (BMIZ), and weight gain velocity (WAZV) were the primary outcome measures.

Mothers completed a self‐report questionnaire including infant and maternal demographic background (maternal age and education, infant sex, and age in weeks), milk feeding at birth (breast or formula milk) and at time of questionnaire (breast, formula, or combination), infant age at commencement of complementary feeding, and method of introducing complementary foods.

To measure the method of introducing complementary foods, participants were asked to estimate their infant's degree of spoon‐feeding at the introduction to complementary feeding. This approach was used as self‐definition of feeding approach (e.g., asking participants if they are following BLW), which varies in reliability (Brown et al., 2017; Brown & Lee, 2011; Cameron, Taylor, & Heath, 2013). Participants were therefore asked to select frequency of spoon‐feeding using a 5‐point scale (*always*, *often*, *sometimes*, *rarely*, and *never*).

### 2.3 Procedure

Participant recruitment posters calling for parents of infants who had started or were about to start complementary feeding (with no mention of approach to complementary feeding) were placed in community halls, family centres, and on social media. Interested participants contacted the lead researcher for further study information. Contact was also made with leaders of local baby groups for permission to attend the group and give a short presentation about the research. Participants were given a study information and consent form, and if they met the inclusion criteria, a suitable time for measuring their baby and completing the background questionnaire was agreed.

Some participants chose to have measurements taken of their baby at just one time point, and these measurements formed a larger cross‐sectional data set. However, participants, particularly those whose baby was as the start of the period of introducing complementary foods (around 4–7 months) were invited to have their baby measured again in at least 4 months' time (towards the end of the complementary feeding period) to form part of a longitudinal data set.

### 2.4 Data analysis

WAZ, LAZ, BMIZ, and WAZV were calculated. WAZ scores were also computed for birth weight. These scores allow infants of different age and sex to be analysed together as they are based on different reference points for each group; *z*‐scores were calculated using the Anthro software package, which uses the World Health Organization (2019) child growth reference standard as the population mean. The *z*‐scores are a standardised index commonly used in research and clinical situations to assess and monitor growth of infants and children (Wang & Chen, 2017). WAZV was calculated as the change in WAZ from birth to age of measurement. This is the measure used in other large cohort studies of infant growth (Azad et al., 2018; Monteiro & Victora, 2005; Sacco, De Castro, Euclydes, Souza, & Rondo, 2013).

Feeding approach was grouped into two main styles by degree of self‐feeding at the start of complementary feeding: predominantly self‐fed (self‐fed always and often) versus predominantly spoon‐fed (self‐fed sometimes, rarely, or never). This was based on similar categorisation used in previous BLW research (e.g., Brown & Lee, 2011; Brown & Lee, 2012; Brown & Lee, 2015) as it allows for occasional spoon‐feeding in predominantly self‐fed infants in contexts such as feeding outside of the home or feeding of a yoghurt.

To examine the impact of method of introduction to complementary foods, two sets of analyses were conducted: one for all infants (the cross‐sectional data set) and one for infants in the longitudinal data set. Where infants had two sets of measurements (at the start and end of the complementary feeding analysis), only one of these sets of measurements was included in the cross‐sectional data set. A decision was made to use the second set of measurements, as this gave a more balanced range of ages across the cross‐sectional data set. Fewer parents were available for measurements when their baby was towards the end of the complementary feeding period (10–12 months) due to practicalities of returning to work.

The association between maternal and infant demographic background and feeding approach was explored. A *t* test was used to examine any differences in birth weight *z*‐score between those who went on to choose a self‐feeding or spoon‐feeding approach. Multivariate analysis of covariance (MANCOVA) was used to examine the difference in WAZ, LAZ, BMIZ, and WAZV between feeding groups. Partial correlations explored associations between infants' age at time of measurement and growth. For infants with two measurements over time, a repeated measures analysis of variance was used to explore differences in WAZ, LAZ, and BMIZ over time.

The combined impact of milk feeding and complementary group upon growth was explored through separate models for self‐fed and spoon‐fed infants, following analyses used in similar studies (e.g., Heinig et al., 1993; Huh et al., 2011). MANCOVA was performed to examine the impact of milk feeding at time of questionnaire in self‐fed and spoon‐fed infants.

For the main sample size, we conducted two separate power calculations (calculated using g‐power) to examine sample size. The first indicated that a sample size of at least 210 would be required to detect a small effect size (*d* = 0.2), with 95% power with alpha at.05. The second, for the repeated measures analyses, indicated that a sample size of 106 was required to detect a small effect size with 95% power, at the.05 alpha level.

Maternal age, education and occupation, infant birth weight, milk feeding at time of questionnaire, and timing of complementary foods were controlled for throughout the analyses.

### 2.5 Ethical statement

Ethical approval was granted from a Department of Psychology Research Ethics Committee. The study adhered to the principles outlined in the Declaration of Helsinki (2018) and the British Psychological Society Code of Research Ethics (2014).

## 3 RESULTS

Two hundred and sixty‐nine infants were weighed and measured. For the infants, 134 were male and 135 were female. Mean age was 42.5 weeks (standard deviation [*SD*] = 9.62; range: 26–59). There were two sets of twins giving a total of 267 mothers. Mean age of the mothers was 31.7 (*SD* = 4.63; range: 21–51). For further maternal demographic details, see Table 1. Six data sets contained missing data for either maternal age (*n =* 4) or maternal education (*n* = 2), giving 263 complete data sets available for analyses, which include these variables.

**Table 1:** Maternal demographic background

	*N*	%
Maternal age		
20–24	13	4.9
25–29	77	28.8
30–34	102	38.2
35–39	58	21.7
40–44	12	4.5
45+	1	0.4
Missing data	4	1.5
Education		
Up to GCSE	20	7.5
A level	25	9.4
Diploma	33	12.4
Degree	113	42.3
Postgraduate	74	27.7
Missing	2	0.7
Parity		
First‐time mother	160	59.9
Second or more	107	40.1

Mean age of starting complementary foods was 23.5 weeks (*SD*: 3.64; range: 10–40 weeks). For feeding approach at the start of complementary feeding, 109 infants were classified as self‐feeding (40.5%) and 160 as spoon‐fed (59.5%). For feeding approach at the time of measurement, 182 infants were predominantly self‐feeding and 86 were being spoon‐fed.

No significant associations were found for either maternal age (*U* = 7,763, *p* = .24) or maternal education, *X* ^2^(4) = 2.28, *p* = .68, and feeding approach.

A significant association was found between milk feeding and complementary feeding approach. Infants who self‐fed were more likely to be breastfed at the time of the questionnaire, and spoon‐fed infants were more likely to be formula fed, *X* ^2^(2) = 46.2, *p* < .000. Infants who self‐fed started complementary foods at a significantly later age (*Mdn* = 26 weeks) than spoon‐fed infants (*Mdn* = 23.5 weeks; *U* = 4,420.5, *p* < .000). Therefore, milk feeding and timing of introduction to solid foods were controlled for throughout.

### 3.1 Feeding approach and infant birth weight

A *t* test found no significant difference in infant birth weight *z*‐score between mothers who went onto follow a self‐fed or spoon‐fed approach, *t*(269) = 0.20, *p* = .84. Infants who self‐fed had a mean birth weight *z*‐score of 0.29 (*SD*: 11) and spoon‐fed a mean *z*‐score of 0.32 (*SD*: 0.97).

### 3.2 Feeding approach and infant growth

A MANCOVA examined differences in WAZ, LAZ, BMIZ, or WAZV between feeding groups. For feeding approach at the start of introduction to complementary foods, no significant difference in WAZ, *F*(2, 254) = 3.34, *p* = .07, BMIZ, *F*(1, 254) = 0.29, *p* = .59, or WAZV (change in weight for age *z*‐score from birth to age at time of measurement), *F*(1, 254) = 1.38, *p* = .24, was found according to feeding approach. However, LAZ significantly differed between the groups, *F*(1, 254) = 6.25, *p* = .01. The spoon‐fed infants had the greatest mean LAZ (*M* = 0.11, *SD*: 1.08), and the self‐fed infants were the shortest (*M* = −0.42, *SD*: 1.20; *p* = .04).

To explore whether growth changed over the period of introduction of solid foods, potentially due to length of exposure to introduction methods, partial correlations between each growth outcome and the length of time exposed to solids (in weeks) were undertaken (controlling for maternal age, education, infant birth weight, and milk feeding type at time of measurement).

Two separate analyses were conducted to explore this association in self‐fed and spoon‐fed infants. Among infants who self‐fed (*N* = 109), no significant associations seen with WAZ (*r* = .023, *p* = .81), LAZ (*r* = .047, *p* = .63), or BMIZ (*r* = −.008, *p* = .94) and length of time exposed to solids. Similarly, among infants who were spoon‐fed (*N* = 160), no significant associations were found for WAZ (*r* = .04, *p* = .62), LAZ (*r* = .07, *p* = .37), or BMIZ (*r* = .008, *p* = .93) and length of time exposed to solids.

### 3.3 Differences in growth over time—Longitudinal analyses

A further subset of infants (*n* = 101) were measured twice: one at the start of complementary feeding and one towards the end with at least 16 weeks between measurements. Mean infant age at first measure was 24.9 weeks (*SD*: 8.12), and mean infant age at second measure was 51.7 weeks (*SD*: 7.47).

Changes in WAZ, LAZ, and BMIZ were examined for the two approach groups over time. One hundred and one infants had two measures (30 self‐fed and 71 spoon‐fed): one at around 6 months and one at around 12 months. Table 2 shows the measurement outcomes and the change over time. Spoon‐fed infants had greater increases in WAZ and BMIZ over time; however, repeated measures analyses of variance did not find these differences to be statistically significant. For WAZ, the between‐subject effect was not significant, *F*(1, 94) = 0.03, *p* = .86, and interaction effect between time and approach group was also not statistically significant, *F*(1, 94) = 1.79, *p* = .18. For LAZ, the between‐subject effect was not significant, *F*(2, 94) = 0.22, *p* = .64, and interaction effect between time and approach group was also not statistically significant, *F*(2, 94) = 0.12, *p* = .73. For BMIZ, the between‐subject effect was not significant, *F*(2, 94) = 1.003, *p* = .32, and interaction effect between time and approach group was also not statistically significant, *F*(2, 94) = 3.34, *p* = .07.

**Table 2:** Longitudinal outcomes: WAZ, LAZ, and BMIZ change over time (*n* = 101)

		Time point 1	Time point 2	Change
Self‐fed	WAZ	−0.04 (1.07)	0.09 (0.94)	+0.13
Spoon‐fed		−0.05 (0.97)	0.24 (0.99)	+0.29
Self‐fed	LAZ	−0.38 (0.72)	−0.39 (0.87)	+0.01
Spoon‐fed		−0.002 (1.02)	−0.03 (1.08)	+0.028
Self‐fed	BMIZ	0.32 (1.11)	0.49 (0.97)	+0.17
Spoon‐fed		−0.08 (1.19)	0.34 (1.13)	+0.42

Abbreviations: BMIZ, body mass index‐for‐age *z*‐score; LAZ, length‐for‐age *z*‐score; WAZ, weight‐for‐age *z*‐score.

### 3.4 Complementary feeding approach, milk feeding, and infant growth

Within each feeding approach group, differences in growth between breastfed and formula‐fed infants were examined. Any breastfeeding (either exclusive or partial) was compared with full formula feeding (Table 3). Any breastfeeding was chosen as it allowed the infant greater opportunity to self‐regulate milk intake, compared with the potential in increased control when infants are solely fed by bottle (Brown et al., 2011). Control variables were maternal age, education, birth weight *z*‐score, and timing of solids introduction.

**Table 3:** WAZ, LAZ, and BMIZ by complementary feeding style and milk feeding group

		Any breastfeeding (either exclusive or partial)	Fully formula fed
Self‐fed	*n*	94	14
Spoon‐fed		74	81
Self‐fed	WAZ: *M* (*SD*)	0.06 (1.00)	−0.07 (1.35)
Spoon‐fed		0.17 (0.98)^*^	0.38 (0.81)^*^
Self‐fed	LAZ: *M* (*SD*)	−0.40 (1.16)	−0.57 (1.44)
Spoon‐fed		0.06 (1.08)	0.15 (1.09)
Self‐fed	BMIZ: *M* (*SD*)	0.49 (0.96)	0.41 (0.97)
Spoon‐fed		0.20 (1.07)	0.41 (0.99)

Abbreviations: BMIZ, body mass index‐for‐age *z*‐score; LAZ, length‐for‐age *z*‐score; WAZ, weight‐for‐age *z*‐score.

* The difference was significant at the.05 level.

Within the self‐fed group, no significant growth differences were found in WAZ, *F*(1, 102) = 0.04, *p* = .84, LAZ, *F*(1, 102) = 0.002, *p* = .88, or BMIZ, *F*(1, 102) = 0.007, *p* = .93, based on whether the infant had received any breast milk (*N =* 94) or were fully formula fed (*N* = 14). However, a significant difference in WAZ was seen among the spoon‐fed infants, *F*(1, 148) = 4.26, *p* = .04. Fully formula‐fed infants (*N* = 81) were found to be significantly heavier (*M* = 0.38, *SD*: 0.81) than infants who had received any breastfeeding (*N* = 74; *M* = 0.17, *SD*: 0.98). No significant differences were seen for LAZ, *F*(1, 148) = 0.28, *p* = .59, or BMIZ, *F*(1, 148) = 2.32, *p* = .13, between full formula feeding and any breastfeeding.

## 4 DISCUSSION

This study explored the impact of method of introducing complementary foods upon growth at 3–12 months, considering further how this might interact with milk feeding method. It found no significant differences in weight or BMI between infants introduced to complementary foods via spoon‐feeding or self‐feeding but found that infants who were spoon‐fed were significantly longer than those who were self‐feeding. However, no significant differences were found for current method of introduction. Moreover, although no significant difference was seen in the growth of infants who were self‐feeding according to milk type, infants who were both spoon‐fed and fully formula fed were significantly heavier than those spoon‐fed alongside being breastfed. The findings have important considerations for how responsive feeding may be a key aspect of growth in the latter part of infancy.

First, infants who were following a traditional complementary feeding approach were longer than those that were self‐feeding. It is possible that method of complementary feeding is affecting length gains due to differences in infant diet. Spoon‐fed infants are more likely to consume fortified infant cereals when compared with self‐feeding infants, especially when introduced to solid foods at an earlier date (Roess et al., 2018). Iron fortified cereals have been linked to increased gains in length‐for‐age at 12 months in Indonesia (Diana et al., 2017), although this finding has not been replicated in developed regions (Pasricha, Hayes, Kalumba, & Biggs, 2013).

However, iron fortified infant foods also contain cow's milk protein or are made up with further added cow's milk or infant formula. High protein intake has been associated with rapid increases in weight and length during infancy (Heinig et al., 1993) and is a risk factor for later overweight and obesity (Hoppe, Mølgaard, Thomsen, Juul, & Michaelsen, 2004; Michaelsen & Greer, 2014).

Although many studies focus just on weight gains, or consider length only in relation to BMI (Rogers & Blissett, 2017), rapid length growth in itself can be a predictor of later obesity (Belfort, Gillman, Buka, Casey, & McCormick, 2013; Elks et al., 2010; Monteiro, Victora, Barros, & Monteiro, 2003). Therefore, it would be interesting for future research to consider the longer term growth trajectories of infants with greater length gains in the early months of life.

However, a number of aspects in the data suggest that infant length may be determining complementary feeding approach. The association we found between spoon‐feeding and longer length was not affected by longer exposure to spoon‐feeding nor was any difference in LAZ seen in the longitudinal analysis. LAZ was associated with method of introducing complementary food but not method at time of measurement. It may be the “thinner” appearance of a longer baby may be prompting the decision to start complementary feeding earlier. Research has shown that concerns over infant weight and energy intake are associated with an earlier introduction of complementary foods (Arden, 2010; Brown & Rowan, 2016), which for infant developmental reasons would require a spoon‐feeding approach. Indeed, in one study, mothers reported that one reason they chose to spoon‐feed was that they were worried their baby wouldn't eat enough (Cameron et al., 2013), whereas mothers who chose a baby‐led approach are less concerned around intake of food (Brown & Lee, 2012).

Neither weight nor BMI differed between the complementary feeding groups, supporting the BLISS study findings (Taylor et al., 2017). There are a number of reasons why a baby‐led approach might not be affecting weight gain. First, the 6‐ to 12‐month period may be too early for differences to emerge, and the percentage of overweight children in the study was small. This may change over time, and one study has shown that pre‐school children who followed a BLW approach were less likely to be overweight compared with those spoon‐fed, although retrospective recall and some self‐reporting of weight limit these findings (Townsend & Pitchford, 2012). Indeed, although in this study longitudinal analysis did not reach significance, the difference in weight change and BMI change was greater in the spoon‐fed group. If these infants followed on the same trajectories, these differences are likely to become significant over time.

Second, as BLW grows in mainstream popularity, the approach may become less “healthy.” Critics of BLW have posited that there is a potential for BLW to allow babies to eat snacks as finger foods such as crisps and biscuits (Cameron, Heath, & Taylor, 2012), and research in larger samples exploring a “looser” version of BLW, where infants self‐fed most of the time, highlighted an increased intake of such snack foods in this group (Rowan, Lee, & Brown, 2019).

However, it might also be that examining method of introducing complementary foods in isolation is not enough, and this is highlighted by differences in weight found in the sample when both milk feeding and method of complementary feeding were considered together. Although no significant difference in the overall sample for weight or BMI was seen between the approach groups, infants who were spoon‐fed and fully formula fed were significantly heavier than those exclusively or partially breastfed, but no impact of milk feeding was seen for self‐feeding infants.

Again, this relationship might be bidirectional. Potentially, experience of both spoon‐feeding and bottle feeding decreases infant opportunity to self‐regulate intake. Breastfed infants typically have more control over their intake of milk as the amount consumed is not visible, whereas formula‐fed infants can be encouraged to finish a bottle (Li, Magadia, Fein, & Grummer‐Strawn, 2012). Mothers who breastfeed are more likely to adopt a responsive feeding approach, letting their infant set the pace of feeds and worrying less around monitoring intake of milk (Brown et al., 2011; Brown & Lee, 2013). Breastfed infants have been identified as better able to regulate their appetite as toddlers (Brown & Lee, 2012) and adolescents (Reyes et al., 2014), although not all studies are conclusive (Hathcock et al., 2014). Additionally, mothers who follow a BLW approach have been shown to be more responsive in their feeding style, showing lower control in terms of restriction, pressure to eat, and monitoring (Brown & Lee, 2011).

Therefore, infants who are both spoon‐fed and bottle fed may have lower opportunity to regulate their own intake compared with infants who can regulate over different meals even if one aspect of their feeding approach (e.g., spoon or bottle) is more “controlling.” This may be affecting weight, although research examining the longer term impact of responsive feeding in infancy is sparse. However, a large body of literature suggests that for older children, a responsive feeding style is associated with the most positive weight outcomes. For example, in a 2015 systematic review of the literature, the authors concluded that restrictive and controlling feeding practices were associated with higher child BMI at ages 4–12 years (Shloim, Edelson, Martin, & Hetherington, 2015).

It is also possible that mothers who are formula feeding and whose infant is already at increased likelihood of being heavier (Dewey, Heinig, Nommsen, Peerson, & Lonnerdal, 1992; Li, Magadia & Grummer‐Strawn, 2012) are drawn to a spoon‐feeding approach. In this study and others, there is a strong association between spoon‐feeding and bottle feeding (Brown & Lee, 2010; Morison et al., 2016; Taylor et al., 2017; Townsend & Pitchford, 2012), potentially due to preferred overall maternal feeding style. Both bottle feeding (Brown et al., 2011) and spoon‐feeding (Cameron et al., 2013) are associated with maternal concerns around intake. Maternal general feeding style appears to be stable over time (Black & Aboud, 2011) with longitudinal research showing that mothers who encouraged their infant to finish their bottle as a baby were more likely to use higher pressure to eat at age 6 (Li, Scanlon, May, Rose, & Birch, 2014). It would be interesting in future research to explore whether mothers who bottle feed but follow BLW were more responsive than average in their bottle feeding approach.

This study does have limitations; participants were older with a higher level of education, and breastfeeding rates were higher than within the general population, although this enabled stronger milk feeding group analyses. Infants were recruited to the research at different ages (around 6–12 months), and thus, the length of time for mothers to recall earlier information (e.g., birth feeding data) was variable, potentially affecting reliability. However, infant feeding data are likely to be accurately recalled within a time period of 3 years (Li, Scanlon, and Serdula (2005), so variability of 6 months is unlikely to have been problematic. Additionally, most infants were considered a healthy BMI (albeit with a broad range of measurements), meaning that analysis of weight groups (underweight, normal, and overweight) would have been underpowered. The small group size of the subgroup with two measurements (longitudinal analyses) was also slightly underpowered, and so these results should be interpreted with caution.

Limitations aside, this study explored a so‐far under‐researched area, highlighting a possible cumulative effect of bottle feeding and spoon‐feeding. It is the first study in the United Kingdom considering method of introducing solid foods to provide prospective and longitudinal measurements. The findings have important implications for the responsive feeding literature and considering infant weight to be a consequence of a cumulation of experiences rather than just one aspect in isolation.

## CONFLICTS OF INTEREST

The authors declare that they have no conflicts of interest

## CONTRIBUTIONS

SWJ was responsible for the study design, data collection, data analysis, draft writing, and critical revisions. ML was responsible for the study design and critical revisions. AB was responsible for the study design, data analysis support, draft writing support, and critical revisions.
